# Impact of lean mass and bone density on glomerular filtration rate estimation in people living with HIV/AIDS

**DOI:** 10.1371/journal.pone.0186410

**Published:** 2017-11-02

**Authors:** Corinne Isnard Bagnis, Laurence Pieroni, Rachida Inaoui, Philippe Maksud, Stéphanie Lallauret, Marc-Antoine Valantin, Roland Tubiana, Christine Katlama, Gilbert Deray, Marie Courbebaisse, Jérôme Tourret, Sophie Tezenas du Montcel

**Affiliations:** 1 Nephrology, AP-HP, Groupe Hospitalier Pitié Salpetrière, Paris, France et Université Pierre et Marie Curie, Paris, France; 2 Biochemistry Department, AP-HP, Groupe Hospitalier Pitié Salpetrière, Paris, France et Université Pierre et Marie Curie, Paris, France; 3 Rhumatology, AP-HP, Groupe Hospitalier Pitié Salpetrière, Paris, France et Université Pierre et Marie Curie, Paris, France; 4 Nuclear Medicine, AP-HP, Groupe Hospitalier Pitié Salpetrière, Paris, France et Université Pierre et Marie Curie, Paris, France; 5 Biostatistics Unit and Clinical Research Unit, AP-HP, Groupe Hospitalier Pitié-Salpêtrière, Paris, France; 6 Infectious Diseases, AP-HP, Groupe Hospitalier Pitié Salpetrière, Paris, France et Université Pierre et Marie Curie, Paris, France; 7 Physiology Department, Hôpital Européen Georges Pompidou, Paris, France et INSERM, Paris, France; 8 Sorbonne Université, UPMC Univ Paris 06 UMR_S1136, Paris, France; 9 Institut Pierre Louis d’EPIdémiologie et de Santé Publique, Paris, France; University Jean MONNET of SAINT-ETIENNE, UNITED STATES

## Abstract

**Context:**

Chronic kidney disease is a frequent complication in persons living with HIV/AIDS. Although previous studies have suggested that the CKD-EPI formula is appropriate to estimate glomerular filtration rate (GFR) in HIV-positive adults with normal kidney function, the optimal way to estimate GFR in those with Stage 3 chronic kidney disease is not known. Moreover, the impact of muscle mass on creatinine level and GFR estimation is unknown.

**Aim and methods:**

Our study aimed to evaluate the accuracy of different diagnostic tests available compared to the gold standard measurement of GFR. A group of 44 HIV-1 patients with an estimated GFR between 60 and 30 ml/min/1.73 m^2^ were included in a single-center cross-sectional study. Serum creatinine and cystatin C were measured. GFR was estimated using Cockcroft-Gault, MDRD, sMDRD, CKD-EPI, CKD-EPIcyst, and CKD-EPIcyst/creat formulae and was measured using isotopic Chrome_51_ EDTA clearance. Bone density and muscle mass were measured by DXA scan.

**Results:**

Mean age was 62±10 years. Mean BMI was 23±4 kg/m^2^. Prevalence of diabetes was 30% and of hypertension was 47%. Viral load was <40 copies/ml for 90% of the patients, and mean CD_4_ count was 446±191 cells/mm^3^. Mean measured GFR was 63.4±16.5 ml/min/1.73 m^2^. All formulae under-estimated GFR. The best relative precision and accuracy were provided by the CKP-EPI formula. sMDRD, CKD-EPIcyst, and CKD-EPIcyst/creat performed worse than the CKD-EPI formula. Body composition did not significantly influence accuracy or precision of GFR estimation.

**Conclusion:**

In HIV-infected patients in stable immunovirologic conditions with CKD stage 3 and high prevalence of metabolic associated conditions, the CKD-EPI formula performed best, although all formulae under estimate GFR.

## Introduction

Chronic kidney disease (CKD) in HIV patients can occur secondarily to viral infections such as human immunodeficiency virus (HIV) and hepatitis C virus (HCV) [1], their treatment, or to metabolic complications. It is now established that around 5% of the patients living with HIV/AIDS (PLWHA) exhibit CKD [2–3]. Estimating glomerular filtration rate (GFR) is part of the critical biological assessment of renal function together with measuring blood pressure and proteinuria and checking for hematuria or leucocyturia [4].

Gold standard measurement of GFR is time consuming, expensive, and a burden for the patients and must be used only in specific clinical situations. Generally, estimation of GFR is sufficient to define work up, follow up, outcome, and drug dosage adaptation. The Cockcroft-Gault formula based on creatinine clearance was used from 1976 [5] until recently to estimate GFR. It is now outdated because weight and age affect the estimate. over and under estimations of GFR occur in over and underweight people, respectively. The Cockcroft-Gault formula is still used for drug dosage adaptation purposes. Recently, several new formulae have been proposed for estimation of GFR in the general population, but each has drawbacks.

Serum creatinine is the main variable used for estimating GFR. Due to inter-assay differences, the prevalence of CKD varies according to the assay used, and standardization is necessary [6]. International guidelines suggest using creatinine-based GFR estimation formula in general population [7]. Although Modification of Diet in Renal Disease (MDRD) is more commonly used in practice, the CKD-EPIdemiology Collaboration (CKD-EPI) equation is the most accurate formula for estimating CKD stage, especially at higher GFR values in the general population [8]. The simplified form of the MDRD (sMDRD) formula uses creatinine, age, and sex with a correcting factor for ethnic groups and also has been updated for the use with standardized creatinine measurements [9]. The CKD-EPI formula is available for creatinine, cystatin C, or both [10, 11]. Choosing the best GFR estimation formula should also take the purpose of the estimation into account (e.g., screening, staging, or drug dosage adapting).

These formulae have previously been tested in PLWHA in two studies (a European cohort and a US cohort) [12, 13] but neither study-included patients with low GFR since mean GFR was around 90 mL/min/1.73 m^2^ in both cohorts. Although performance of the CKD-EPI formula has been well characterized in patients with high GFR, accuracy is not known in PLWHA and CKD stage 3 patients. Moreover, our study evaluated the impact of body composition (particularly lean mass) on the performance of the GFR estimates.

All formulae are based on serum creatinine, but the validity of creatinine measurement in the HIV population has not been determined. Serum creatinine serves as a reliable muscle mass biomarker in general population as well as in PLWHA. Attendant to the major increase in lifespan in HIV patients due to available therapies are signs of premature aging with notable changes in the musculoskeletal system. Imaging studies such as dual-energy X-ray absorptiometry (DXA) are among the gold standard methods for assessing body fat and lean body mass (LBM), approximately half of which is made of skeletal muscle mass. Deficient skeletal muscle mass defines sarcopenia [14]. Impaired muscle function is a common finding in PLWHA that has gained less attention than lipodystrophy. Therefore, the impact of lean mass on GFR estimation in PLWHA is not known.

Our study compared a gold standard measurement of GFR (mGFR) to different recommended estimation formulae (Cockcroft-Gault, sMDRD, and CKD-EPI) using standardized creatinine, cystatin C, or both and evaluated the impact of lean mass changes on the reliability of GFR estimation.

## Material and methods

### Study design

PLWHA were included in a single-center cross-sectional study (Pitié Salpetrière Hospital, Paris, Assistance Publique) designed to evaluate the accuracy of the different diagnostic tests available compared to the gold standard measurement of GFR. Inclusions ran from June 22, 2009 to March 8, 2012. The main objective was to compare measured and estimated GFR. Secondary objectives were to highlight the impact of body composition changes on measurement and estimation of GFR (S1 File, S2 File and S3 File).

### Patients

Patients followed at our HIV renal unit were evaluated for eligibility to participate in the study. Adult (> 18 years), male, Caucasian patients exhibiting an estimated GFR (eGFR) based on Cockcroft-Gault between 60 and 30 ml/min were included (Fig A in S1 Fig). We chose to include patients with stage 3 CKD, as earlier stages are less critical in terms of prevention and nephroprotective strategies. Moreover, there are few patients with CKD stage 4 and 5 in our active file. Acute renal failure, thyroid disease, steroids therapy, or inability to undergo Dexascan or venous blood catheterization was exclusion criteria. At the time of inclusion, all patients had to have been in a steady state for at least 6 months with regard to eGFR, ART regimen and HIV disease markers.

The research was conducted in accordance with the guidelines and under the terms of the Declaration of Helsinki. The local board (CPP- Ile de France VI Groupe Pitie Salpetriere N°79–08) approved the study protocol. All patients signed informed consent after receiving clear and intelligible explanations of the study goals and requirements. The study was authorized by AFFSAPPS under N°P080202/N° ID RCB 2008-A00445-50 and published on the Clinical Trials website under the number P080202.

### Estimation and measurement of GFR and body composition assessment

Serum creatinine was measured using an IDMS-traceable assay (compensated colorimetric method from Roche Diagnostics) for CKD-EPI, CKD-EPIcyst, and CKD-EPIcyst/creat GFR estimation. An enzymatic method using CreaPlus on a Roche analyzer was used for other formulae GFR estimations. Urea, albumin, proteinuria, and cystatin C were measured during the patient’s visit in our ambulatory unit using a BN Prospec nephelometer from SIEMENS.

eGFR was estimated using Cockcroft-Gault, sMDRD, CKD-EPI, CKD-EPIcyst, and CKD-EPIcyst/creat [5, 9, 10–12]). eGFR was measured through a continuous ^51^Cr-ethylene-diamine tetra-acetic acid (^51^Cr-EDTA, GE Healthcare) infusion method performed in our nuclear medicine department. A priming dose of 0.5 μCurie/kg body weight of ^51^Cr-EDTA was injected intravenously, followed by a constant ^51^Cr-EDTA infusion. After allowing 1 hour for equilibration of the tracer in the extracellular fluid, urine was collected and discarded. Average renal ^51^Cr-EDTA clearance was assessed during six consecutive 30-min clearance periods. Blood was drawn at the midpoint of each clearance period with the last collection 300 min after injection of the priming dose. The radioactivity measurements in 1-mL plasma samples and in urine samples were carried out on a Packard Cobra 3-inch crystal γ-ray well counter.

All GFRs (whether estimated or measured) were indexed to actual body surface area.

Body composition was measured by biphotonic absorptiometry DXA scan to estimate lean mass in each patient. DXA is one of the gold-standard techniques in the analysis of body composition, providing assessment and quantification of fat mass, lean mass, and bone mineral content. Only total body T-scores were available. A T-score below -1 was used as a proxy for osteoporosis.

### Statistical analysis

Means +/- SD and medians (IQR) were used for quantitative values. For binary or quantitative values, frequency and prevalence values were used. The predictive performance of the five formulae was assessed using the following parameters:

- Absolute bias: defined as the mean difference between mGFR and eGFR; a positive value means that eGFR under-estimates true GFR
- Relative bias: calculated as absolute bias/mGFR x 100
- Precision: evaluated by the standard deviation of the mean difference between eGFR and mGFR (absolute and relative)
- Accuracy: defined as the proportion of eGFR values within ± 30% of the mGFR
- Agreement: evaluated by the Bland and Altman method

Pairwise accuracies were compared using McNemar’s exact test. In addition, weighted kappa values were computed.

To decrease variability, only Caucasians patients were included. When the maximal absolute bias was set at 3 ml/min/1.73 m^2^ and with an expected precision < 20 ml/min/1.73 m^2^, 60 patients needed to be included to obtain a power of 81% (alpha 5%) [14].

Statistical analyses were performed using the SAS 9.2 statistical package (SAS Institute Inc.).

Data were collected and analyzed in our hospital.

## Results

### Patient population

Forty-five patients were selected and included based on their latest available value for GFR (less than three months prior) estimated with Cockcroft-Gault formula. Mean values were 51.9±13.1 (sMDRD). One patient did not come to the second visit and 44 patients completed the study.

Mean age was 62±10 years with 82% older than 50 years. The mean time from HIV diagnosis was 19±7 years. Mean BMI was 23±4 kg/m^2^; 25% of the cohort had a BMI greater than 25. Prevalence of diabetes was 30%, and hypertension prevalence was 47%. Viral load was less than 40 copies/ml for 90% of patients, and mean CD_4_ count was 446±191 cells/mm^3^. Demographic data, treatment details, mean blood and urine biological data are shown in Tables 1 and 2.

**Table 1:** Demographic data and treatment details at inclusion.

Variable	Mean Or frequency (SD or %)
Age	62 (10)
Age >50 (n = 44)	36 (82%)
Time since HIV infection diagnosis (years) (n = 43)	19 (7)
Diabetes mellitus	13 (30%)
Hypertension (n = 36)	17 (47%)
HAART	42 (95%)
Tenofovir	20 (45%)
Atazanavir	15 (34%)
CD_4_ count (cells/mm^3^) (n = 38)	446 (191)
*CD*_*4*_ *count (cells/mm*^*3*^*) < 350 (n = 38)*	13 (34%)
Viral load (n = 40)	
*≤40 copies/ml* *≤20 copies/ml*	36 (90%) 28 (70%)
*72* copies/ml	1 (2.5%)
* 120* copies/ml	1 (2.5%)
* 160* copies/ml	1 (2.5%)
* 56*,*402* copies/ml	1 (2.5%)
HBV infection	10 (23%)
HCV infection	4 (9%)
HCV/HBV co infection	1 (2%)

HBV: hepatitis B virus; HCV: hepatitis C virus; HAART: highly active anti-retroviral therapy.

**Table 2:** Biological data.

Variable	Normal values	Mean Or frequency (SD or %)
mGFR (ml/min/1.73 m^2^)		63.4 (16.5)
**Blood**		
* *Enzymatic plasma creatinine (μmol/L)	According to age and sex	139.9 (51.5)
* *Urea (mmol/L)	According to age and sex	8.8 (3.1)
* *Albumin (g/L)	37–48	44.4 (3.7)
* *CRP (mg/L)	< 5	7.1 (8.1)
* *CRP>10 mg/L		6 (14%)
* *Cystatin C (mg/L)	< 1.2	1.6 (0.5)
**Urine**		
* *Creatinine (mmol/L)	ND	9.0 (5.2)
* *Cystatin C (mg/L)	ND	1.0 (4.7)
* *Proteinuria (g/L)	<0.10	0.5 (0.7)

mGFR: measured glomerular filtration rate; CRP: C-reactive protein; GFR: glomerular filtration rate.

### GFR estimation and formulae performances

Mean measured isotopic GFR was 63.4±13.5 ml/min/1.73 m^2^. All formulae under-estimated GFR. Best performance was provided by CKD-EPI with accuracy of 30%, good precision, low bias, and the highest correlation coefficient of all formula tested. CKD-EPIcyst and CKD-EPIcyst/creat performed more poorly than the CKD-EPI and sMDRD in estimating GFR (Table 3).

**Table 3:** Predictive performance of GFR estimation formulae based on creatinine, cystatin C, or both.

Measured GFR	63.39±16.47					
GFR Estimate	Mean ± SD	R (Pearson Coefficient)	Bias (ml/min/1.73 m^2^)	Relative Bias (%)	Absolute Precision (ml/min/1.73 m^2^) Relative precision (%)	Accuracy 30%
**Cockcroft Enzymatic**	53.14 ± 18.69	0.62**	10.3**	15.0*	15.4 (24.8)	70
**Cockcroft Enzymatic/** **Body surface area**	57.52 ± 24.43	0.52*	5.9	7.9	21.2 (35.0)	64
**MDRD Enzymatic**	49.66 ± 14.57	0.66**	13.7**	20.3**	12.9 (18.5)	75
**CKD-EPI**	51.82 ± 16.29	0.70**	11.6**	17.3**	12.8 (19.2)	82
**CKD-EPIcyst**	48.84 ± 18.62	0.51*	14.5**	21.7**	17.5 (25.1)	61
**CKD-EPIcyst/creat**	49.66 ± 16.51	0.64*	13.7**	20.7**	14.0 (20.1)	68

*: P<0.001

**: p<0.0001; GFR: glomerular filtration rate expressed as ml/min/1.73 m^2^.

### Body composition

Whole body bone mineral density (BMD) analyses were performed with the aim of analyzing the impact of lean mass on the performance of the different estimation formulae. Mean normal values for lean mass are not known in PLWHA, but in healthy persons weighing 70 kg it has been proposed that the expected values of lean mass should be around 80% of the total body composition [15]. PLWHA have been shown to exhibit lower muscle mass and higher frequency of sarcopenia than healthy subjects [16]. We defined three groups of patients depending on their relative lean mass: i) below 70% of total mass, ii) between 70% and 85%, and iii) above 85%. Of the 44 patients in the cohort, only 16 exhibited muscular mass values in normal to low range. Using total body BMD T-score, 25 patients (57%) exhibited values below -1 SD (Table 4). We then divided the patients into three groups according to relative value of lean mass. Lean mass values decreased non-significantly with age as expected. Patients with a relative lean mass value below 70% had higher BMI (p = 0.0005) and measured weights (p = 0.0024). They also had the lower values for T-score (often below -1) (p = 0.0017) (Table 4).

**Table 4:** Body composition.

Variable	Mean Or frequency	SD or %	Min	Max
Measured weight (kg)	70	14	42	105
Height (cm)	173	9	150	192
BMI	24	4	17	32
<18.5	2	5%		
[18.5–25[	31	70%		
[25–30[	5	11%		
≥30	6	14%		
Total body mass (kg) (sum of values of body compartments by DXA)	71	13	42	105
Lean mass (g)	53 872	7 361	35 232	65 241
Lean mass (%)				
< 70%	10	23%		
> 85%	6	14%		
Fat mass (g)	14 481	7 953	3 831	35 577
Fat mass (%)	19.4	7.4	7.8	35.7
BMC (g)	2 674	538	1 486	4 114
Bone mass (%)	3.8	0.5	2.6	5.0

BMI: body mass index. BMC: bone mineral content.

### Impact of body composition on GFR estimation

We evaluated whether lean mass and bone mineralization could influence GFR estimations (Tables 5 and 6, respectively). The Cockcroft-Gault formula estimated GFR was significantly correlated with lean mass values and bone mineralization (T-scores). No significant difference was observed based on lean mass or T-score categorization.

**Table 5:** Patients characteristics and GFR estimation in patients grouped by lean mass.

Variable	Median (IQR) or frequency (%) in each lean mass category: N (%)			
	<70% (N = 10)	70–85% (N = 28)	>85% (N = 6)	P-value†
Lean Mass		57961 (52716–60841)	55052 (47658–58638)	51475 (44325–61990)	-
Serum creatinine (enzymatic)	124 (118–143)	127 (119–154)	121 (114–131)	0.7425
Age >50 years	9 (90%)	23 (82%)	4 (67%)	0.6271
Weight (kg)	86 (79–94)	70 (61–73)	59 (51–72)	0.0024
BMI (kg/m^2^)	30 (25–31)	23 (21–24)	21 (18–21)	0.0005
BMI > 25 kg/m^2^	6 (60%)	5 (18%)	0	0.0132
Low T-score (<-1)	1 (10%)	19 (68%)	25 (83%)	0.0017
Years since HIV diagnosis	19 (16–20)	22 (16–24)	21 (18–23)	0.4588
CD_4_ < 350/mm^3^	3 (33%)	8 (35%)	2 (33%)	1
Tenofovir	2 (20%)	14 (50%)	4 (67%)	0.1637
Atazanavir	3 (30%)	11 (39%)	1 (17%)	0.6586
GFR measured	57 (53–72)	66 (53.5–70.5)	64.5 (50–74)	0.8653
Cockcroft Enzymatic	68 (53–75)	53 (40–58.5)	58 (34–59)	0.0488
Cockcroft Enzymatic/SC	81 (56–87)	54 (39.5–64)	55.2 (32–65)	0.0310
MDRD Enzymatic	51 (43–54)	50.5 (39.5–54)	53.5 (48–59)	0.6856
CKD-EPI	51.5 (44–57)	51.5 (40–56.5)	57 (49–65)	0.5805
CKD-EPIcyst	46.5 (39–55)	50 (33.5–56)	51.5 (32–60)	0.9462
CKD-EPIcyst/creat	50 (42–54)	51.5 (33–58)	54 (40–64)	0.8605

† Kruskal-Wallis test for quantitative variables and Fisher exact test for qualitative variables

BMI: body mass index, GFR: glomerular filtration rate **expressed as ml/min/1.73 m^2^.**

**Table 6:** GFR estimation in patients grouped according to bone mineralization.

	N (Median (IQR) or N (%) in each T-score category		
Variable	< -1 (N = 25)	≥ -1 (N = 19)	P-value †
Age >50 years	20 (80%)	16 (84%)	1
Weight (kg)	68 (57–72)	79 (70–90)	0.0012
BMI (kg/m^2^)	21 (21–23)	25 (22–30)	0.0010
BMI > 25 kg/m^2^	2 (8%)	9 (47%)	0.0045
Measured GFR	66 (53–71)	59 (53–72)	0.8682
Years since HIV diagnosis	20 (14–24)	20 (17–23)	0.9608
CD_4_ count < 350/mm^3^	7 (33%)	6 (35%)	1
Tenofovir	11 (44%)	9 (47%)	1
Atazanavir	11 (44%)	4 (21%)	0.1985
Cockcroft Enzymatic	52 (35–59)	60 (51–73)	0.0380
Cockcroft Enzymatic/body surface area	49 (37.5–61)	68 (50–84)	0.0310
MDRD Enzymatic	50 (47–54)	51 (41–55)	0.8309
CKD-EPI	52 (48–60)	54 (44–57)	0.8775
CKD-EPIcyst	48 (35–53)	51 (36–60)	0.3933
CKD-EPIcyst/creat	49 (40–60)	52 (39–59)	0.6019

† Wilcoxon two-sample test for quantitative variables and Fisher exact test for qualitative variables

BMI: body mass index; GFR: glomerular filtration rate expressed as ml/min/1.73m^2^.

Bias in estimated GFR was evaluated as a function of patient characteristics (Table 7 and S2 Fig). Cockcroft-Gault bias was significantly lower in patients with low bone mineralization than in the other two groups, indicating less over estimation of GFR. Other formulae had no significant difference in accuracy or bias as a function of bone mineralization. Low bone mass (estimated by T-scores below 1 SD) was significantly correlated to increased lean mass. No other factor than BMI significantly impacted T-scores. The treatment received (vitamin D, calcium, bisphosphonates) had no significant impact on bone mass. In our study, tenofovir did not exhibit any significant effect on lean mass nor bone mass but significantly influenced absolute bias (p<0.05): A significant difference was observed between patient treated and not treated with tenofovir with regard to bias when sMDRD was used. No significant difference was observed with atazanavir.

**Table 7:** Absolute bias based on clinical and biological characteristics.

Variable		N	Cockcroft Enzymatic	Cockcroft Enzymatic/SC	MDRD Enzymatic	CKD-EPI	CKD-EPIcyst	CKD-EPIcyst/creat
Age (years)	≤ 50	8	9 [-1.5; 18.5]	7.1 [-17.9; 18.4]	18.5 [13; 25]	15 [8; 22.5]	15 [-6.5; 29.5]	16 [4; 25]
	> 50	36	10 [1.5; 18]	7 [-5.5; 17.7]	9 [3.5; 21]	9 [1.5; 18.5]	16.5 [3.5; 22.5]	12.5 [3; 24.5]
BMI	< 25	33	13 [5; 19]*	8.9 [3.1; 20]	9 [5; 23]	9 [3; 19]	17 [2; 22]	12 [3; 21]
	≥ 25	11	-2 [-12; 10]*	-8.3 [-28.8; -0.4]	13 [8; 31]	10 [8; 27]	16 [1; 38]	13 [3; 33]
T-score	Low (<1SD)	25	16 [7; 21]*	13.6 [5.3; 21.3]	14 [5; 24]	11 [3; 20]	17 [2; 25]	14 [5; 26]
	Normal (>1SD)	19	4 [-4; 10]*	-1.7 [-27.2; 7.5]	11 [7; 28]	9 [6; 24]	14 [-3; 21]	11 [3; 23]
Lean Mass (%)	< 70	10	3.5 [-12; 8]*	-5 [-27.2; 0.1]	8.5 [3; 13]	9 [2; 11]	10.5 [-3; 20]	10.5 [3; 17]
	≥ 70	34	12.5 [5; 21]*	9.2 [2.4; 23.9]	12.5 [5; 24]	11 [4; 20]	17 [2; 25]	13.5 [3; 26]
CD_4_ count	< 350	13	10 [5; 18]	10.7 [-8.1; 21.3]	12 [5; 18]	11 [5; 17]	5 [-3; 21]	11 [3; 21]
	≥ 350	25	11 [3; 18]	9.2 [1.6; 21.3]	13 [7; 26]	11 [6; 25]	20 [10; 28]	16 [10; 29]
TDF	No	24	10.5 [1.5; 18]	-5 [-27.2; 0.1]	8.5 [2; 16.5]*	9 [-1; 15.5]	13.5 [-0.5; 20.5]	10.5 [3; 19]
	Yes	20	9.5 [-1.5; 18.5]	6.5 [-3.7; 16.9]	15 [8.5; 27.5]*	12 [5.5; 25.5]	19.5 [3; 36]	15 [8.5; 31.5]
ATZ	No	29	10 [0; 16]	7.5 [-1.7; 21.3]	9 [3; 17]	8 [1; 16]	17 [-3; 23]	13 [1; 21]
	Yes	15	11 [-2; 28]	7.2 [-2.1; 19.2]	16 [7; 26]	15 [9; 25]	11 [5; 32]	11 [7; 29]

*: P<0.05 (Wilcoxon 2 sample test t approximation); BMI: body mass index, TDF: tenofovir; ATZ: atazanavir.

We compared accuracies of GFR estimates: None of the comparisons were significant, although trends were observed for the comparisons between CKD EPI and CKD-EPI Cyst (p = 0.0965) and between CKD EPI and CKD-EPI Combined (p = 0.070). Three coefficients were higher than 0.70: Cockcroft Enzymatic versus Cockcroft Enzymatic /body surface area (0.78 [0.61;0. 96]), MDRD Enzymatic versus CKD EPI (0.80 [0.59; 1]), and CKD EPI-Cyst versus CKD EPI-Combined (0.76 [0.57;0. 95]). (S3 Fig)

## Discussion

Our study focused on comparing different GFR estimation formulae to determine accuracy in HIV-infected patients with stage 3 CKD. Only Caucasian, male patients with a long exposure time to HIV infection and treatments and eGFR between 30 and 60 ml/min/1.73 m^2^ estimated using routine estimation strategies were included. The mean age in our cohort is higher than that of the 2011 French PLWHA cohort (mean age 48 years) [15]. Both age and duration of exposure to HIV are known risk factors for CKD. Not surprisingly, prevalence of diabetes and hypertension were also significantly higher than national prevalence estimates (9% and 26%, respectively) [17]. Immuno virologial control seems to be within the national range (HAART treated patients = 95%; undetectable viral load = 90%). Exposure to tenofovir was 45%, much lower than usual (around 70% of the patients treated in a 2011 French cohort [18]) probably due to CKD (all included patients had been referred to the renal clinic for CKD). About 34% of patients were exposed to atazanavir, a higher proportion compared to national range of 14% reported in the 2011 study [18]). We found that 9% of patients were co-infected with HCV and 23% with HBV, which is much lower for HCV and much higher for HBV than the national numbers (16.5 to 18.9% for HCV depending of the cohorts studied and 7% for HBV in 2004). In our cohort, 14% of the patients exhibited some level of chronic inflammation with a CRP above 10 mg/L.

Our data indicate that all formulae under-estimate GFR, with the best performance afforded by the CKD-EPI formula. There was no benefit in using complex formulae such as those combining creatinine and cystatin C. We showed no effect of lean mass or bone density on the performance of GFR estimation. Only the Cockcroft-Gault formula was more precise in low bone mineral density/high lean mass patients probably because, in this case, weight is a marker of the lean mass. Therefore, in HIV-infected patients, our study shows that CKD-EPI provides the best performance and that this method of estimation is not influenced by body composition or tenofovir use. These results support the American Guidelines for GFR estimation in HIV-infected patients [4], even in patients with GFR under 60 mL/min/1.73 m^2^, who were under represented in previous studies [19, 20].

Our study has drawbacks. It is underpowered because of the small number of included patients (n = 44); (60 patients were needed). Our study did not include women or patients of African descent because, due to expected number of patients needed, we deliberately chose to restrain our study to Caucasian men to avoid potentially confounding factors of gender and ethnicity.

The healthy value for lean mass is not clear in the literature [21], and very few studies have focused on the optimal strategy to estimate adult lean mass. DXA scan appears to be an accurate method to estimate lean mass [22, 23] although no recent study documents the best method. No reliable data on lean mass are available in PLWHA except one recent study showing increased prevalence for sarcopenia in HIV patients and suggested that lean value could be reliably estimated using DXA [24]. Our study showed that differences in lean mass and bone mass do not influence GFR estimation.

Surprisingly, tenofovir treatment resulted in a significant impact on absolute bias in enzymatic MDRD; clinically relevance is unclear and should be further explored. Moreover, our study is underpowered to support any conclusions on the impact of drug on bone mass.

Our study also has strengths. Very few studies have documented compared GFR estimation methods to gold standard measurements. Indeed, most studies compared different equations without using a standard measurement, which does not provide information with regard to the validity of the GFR estimations [25, 26]. Berenger et al. [27] studied only 22 patients with various degrees of stable kidney function and DXA lean body mass assessment were included. These authors used as a reference a single dose of intravenous iothalamate. Margolick et al. [28] measured GFR with an iohexol-based assay in 455 HAART-treated PLWHA with normal renal function but did not compare reliability to estimated GFR formulae. Bhasin et al. [29] did explore performance of GFR estimation formulae by comparison to measurements made using iohexol in HIV-infected patients with normal renal function. They found that HIV treatment factors and markers of T cell activation significantly impacted reliability of cystatin-based GFR estimation. Wyatt et al. [30] also studied iohexol-measured GFR in an ART-naïve Kenyan population with normal renal function.

What the gold standard should be is debated. Levey et al. recently discussed how bias and precision of eGFR compared to mGFR may be affected by the GFR measurement method used for developing the estimating equation and emphasized the complexity of GFR estimation and measurement [31]. The bias of various measurement methods seems relatively small and imprecision can be reduced by adherence to standardized protocols, providing accuracy substantially greater than that of eGFR [32].

Currently, serum creatinine measurement is the mainstay of routine laboratory monitoring of renal function despite influences of by lean mass on the accuracy. Since PLWHA may suffer from decreased lean mass, we hypothesized that a drop in lean mass would interfere with GFR estimation performance using validated formulae. The Cockcroft-Gault formula, because of its higher accuracy relative to other formulae, could be advantageously used in PLWHA when eGFR is needed to guide drug dosage decisions.

Cystatin C, a cysteine proteinase inhibitor, has been proposed as a more reliable GFR marker than creatinine. It is freely filtered across the glomerular membrane and is metabolized in proximal tubules [33]. Recent studies suggest that it is a more sensitive and reliable GFR marker and a stronger predictor of the risk of death and cardiovascular events than serum creatinine [34]. However, cystatin C is influenced by factors such as age, body mass index, smoking, and levels of C reactive proteins, inflammation, hypertension, and cancer. Several equations to estimate GFR from serum cystatin C have been developed in the last few years, and it has been argued that they have better reliability than those based on creatinine because of a lack of influence of lean mass. There is no consensus in the community with regard to use of this formula in non-HIV patients, and little experimental data are available in the HIV population [35]. Gupta et al. [36], analyzed variations of cystatin C- and creatinine-based formulae in naïve and treated patients and concluded that “antiretroviral-associated changes in renal function vary in magnitude and direction based on the estimating equation used”.

Barraclough et al. [37] studied 22 patients (male and Caucasians with a normal GFR) and compared the Cockcroft-Gault formula to a simplified MDRD and to cystatin C formulae versus the technetium-99 gold standard measurements. The CKD-EPI formula, which would have been a more appropriate choice with regard to the patients mean GFR (91 ml/min/1.73 m^2^), was not studied in this article. In the DART trial, Störh et al. [38] compared the Cockcroft-Gault and MDRD formulae in 3,316 adult PLWHA in Africa but no clear conclusions could be drawn in absence of a gold standard measurement. The largest study to date with a gold standard measurement was conducted by Inker et al. [19]. The authors evaluated the performance of the MDRD and CKD-EPI-creatinine 2009, CKD-EPI-cystatin C 2012, and CKD-EPIcyst/creat GFR estimates with GFR measured using plasma clearance of iohexol in 200 PLWHA on stable antiretroviral therapy. Creatinine and cystatin C assays were standardized to certified reference materials. Mean value for measured GFR was 87 ml/min/1.73 m^2^. The cystatin C/creatinine equation was significantly more accurate than other formulae. These authors therefore concluded that routine evaluation of GFR in HIV patients with normal renal function could be performed using creatinine based CKD-EPI equations as in the general population. Our study supports this conclusion.

In contrast to these studies, it is worth noting that Gagneux-Brunon et al. [35] observed very low accuracies of GFR estimates in patients with GFR < 60 ml/min/1.73 m^2^ and that Inker [19] also showed lower accuracies than we observed. Both authors also observed that eGFR underestimates true GFR.

The complexity of scientific and reliable assessment of GFR must not overshadow the goal of classifying renal filtration rate value for a given patient. The aim of GFR determination is to estimate the risk for cardiovascular and renal further events. The required precision to provide optimal care is not well defined. The current data suggests that GFR can be estimated using routine equations in PLWHA as in the general population. It is important to offer regular monitoring and to identify and treat impaired renal function when necessary to moderate cardiovascular and renal risks.

## Supporting information

S1 File Research project Sidaction. (DOC)

S2 File Hiver’s study protocol. (DOCX)

S3 File Trend statement for HIVERS study. (PDF)

S1 Fig Flow chart of the study (Consort). (PDF)

S2 Fig Fig 1: Accuracy of estimating equations versus measured glomerular filtration rate for 7 clinical and biological characteristics. Accuracy: percentage of estimates more than 30% of measured GFR.
(PDF)

S3 Fig Fig 2: Bland et Altman plots of estimating equations versus measured glomerular filtration rate. The red dashed line correspond to the complete agreement, the black solid line to the mean observed absolute bias, the black dashed lines to the 95% confidence interval of the mean bias and the dashed blue lines to the 95% limits of agreement.
(DOCX)
