# Supplementary material for: Impact of lean mass and bone density on glomerular filtration rate estimation in people living with HIV/AIDS
Source: PLoS One. 2017 Nov 2;12(11):e0186410. doi: 10.1371/journal.pone.0186410 (PMC5668131; doi:10.1371/journal.pone.0186410)
Supplement: S1 File — (DOC) [file pone.0186410.s001.doc]

##

## **Marqueurs non invasifs du debit de filtration glomérulaire chez le sujet infecté par le VIH – influence de la composition corporelle**

**Etat des connaissances sur le sujet et références bibliographiques.**

***L’insuffisance rénale chronique est fréquente chez le sujet infecté par le VIH***

L’infection par le VIH étant désormais une maladie chronique, la prévalence des complications, en particulier de l’insuffisance rénale chronique (IRC) devient une préoccupation majeure. Tout d’abord le traitement par HAART n’a pas fait disparaître l’atteinte rénale spécifique du VIH ou HIVAN qui est responsable de 40 à 60% des atteintes rénales histologiques dans des études récentes (Szczech, Gupta et al. 2004). De plus, d’autres néphropathies sont observées chez ces patients en particuliers celles liées aux co-infections virales dont la prévalence est toujours très significative. L’insuffisance rénale chronique terminale n’est plus exceptionnelle dans cette population et le nombre de patients dialysés et infectés par le VIH augmente aux Etats Unis et en Europe(Tourret, Tostivint et al. 2006). En dehors du rôle spécifique du virus, le sujet infecté par le VIH présente un grand nombre de facteurs de risque d’IRC non spécifiques comme l’âge, l’hypertension artérielle, le diabète non insulino dépendant, l’exposition à des traitement médicamenteux multiples et prolongés(Krawczyk, Holmberg et al. 2004), les épisodes antérieurs d’insuffisance rénale aigue(Wyatt, Arons et al. 2006)…

L’amélioration considérable de la survie des patients infectés par le VIH explique le vieillissement constant de cette population. Le nombre de sujets infectés âgés de plus de 65 ans a été multiplié par 10 en 10 ans et on estime qu’en 2015, 50% des patients vivants avec le virus auront plus de 50 ans(Gebo 2006). En France actuellement, un homme sur 4 et une femme sur 8 vivant avec l’infection ont plus de 50 ans (Yeni 2006). De plus, le nombre de contaminations chez des sujets de plus de 50 ans est en augmentation(Gebo 2006) atteignant 19% en France (Yeni 2006).

La prévalence de l’hypertension artérielle est évaluée à 7 à 29% chez le patient infecté par VIH (Jung, Bickel et al. 2004; Seaberg, Munoz et al. 2005; Crane, Van Rompaey et al. 2006) et même jusqu’à 42% chez le sujet de plus de 55 ans (Shah, McGowan et al. 2002).

L’insuffisance rénale aigue (IRA) est une complication fréquemment observée chez le sujet infecté par le VIH. Sa prévalence atteint 5.9% années-personnes dans un étude récente portant sur 754 patients ambulatoires traités par HAART (Franceschini, Napravnik et al. 2005) et 6% chez le patient hospitalisé infecté par le VIH comparé à 2.7% chez le sujet séronégatif (Wyatt, Arons et al. 2006). L’âge, le diabète, l’IRC pré-existante, l’atteinte hépatique et les co-infections virales sont de puissants facteurs de risque d’IRA. L’IRA est un facteur de risque majeur de mortalité hospitalière et d’augmentation de la durée de l’hospitalisation. Le fait d’avoir présenté un épisode d’IRA favorise la persistance d’un certain degré d’IRC.

Ainsi, la prévalence de l’IRC dans différentes populations de sujets infectés par le VIH (traités ou non, contrôlés ou non) peut atteindre de 5 à 25% (Choi, Rodriguez et al. 2006; Cheung, Wong et al. 2007; Mocroft, Kirk et al. 2007; Morlat, Déti et al. 2007) suivant la méthode choisie et la définition utilisée.

***L’insuffisance rénale chronique doit être dépistée tôt, évaluée précisément et prise en charge de façon adéquate (néphroprotection, adaptation des prescriptions à la fonction rénale, prise en charge des autres facteurs de risque cardiovasculaires) afin de ralentir la dégradation de la fonction rénale des patients, diminuer la morbi-mortalité cardiovasculaire et le risque d’évolution vers le stade SIDA.***

De nombreuses études ont montré l’intérêt de la prise en charge précoce des néphropathies dans d’autres populations que le sujet infecté par le VIH(Locatelli, Marcelli et al. 1996). Dans la néphropathie diabétique, un bénéfice sur la vitesse de dégradation de la fonction rénale et sur la morbi-mortalité cardiovasculaire a été démontré en cas de prise en charge aux stades les précoce de l’atteinte rénale (I et II de la classification de la NKF)(Parving, Lehnert et al. 2001) après mise en évidence d’une néphropathie incipiens reposant sur l’existence d’une microalbuminurie.

Chez les patients atteints d’HIVAN, néphropathie spécifique du virus, il a été démontré expérimentalement que le traitement par les inhibiteurs de l’enzyme de conversion de l’angiotensine a un effet favorable sur l’évolution de la fonction rénale et de la protéinurie(Hiramatsu, Hiromura et al. 2007). Les études cliniques, bien que non randomisées et portant sur de petits nombres de patients, montrent également un bénéfice des IEC chez les sujets atteints d’HIVAN et présentant une protéinurie(Kimmel, Mishkin et al. 1996; Wei, Burns et al. 2003).

L’insuffisance rénale chronique est associée chez le patient infecté par le VIH à un moins bon pronostic avec évolution plus fréquente vers le stade SIDA et une augmentation de la mortalité(Szczech, Gange et al. 2002; Gupta, Mamlin et al. 2004; Szczech, Gupta et al. 2004).

Chez le sujet infecté par le VIH et dialysé nous avons montré que la survie à deux ans est comparable à celle d’une cohorte de patients non VIH, non diabétiques âgés en moyenne de 20 ans de moins(Tourret, Tostivint et al. 2006). Nous avons également montré que les prescriptions d’antirétroviraux font l’objet d’erreurs liées soient à la crainte de surdosage (avec sous dosage fréquent en inhibiteurs de protéases qui ne nécessitent pas d’adaptation chez le dialysé) soit au maintien d’une dose normale (avec surdosage et risque d’effets secondaires). Le contrôle non optimal de l’infection virale chez le dialysé pourrait expliquer les résultats démontrés sur la survie, les facteurs de risques indépendants de mortalité chez ces patients étant un taux bas de CD4 et une charge virale élevée.

Compte tenu du risque cardiovasculaire global du patient infecté par le VIH, le diagnostic précoce d’une altération de la fonction rénale est important mais le retentissement sur le pronostic cardiovasculaire d’une intervention précoce n’est pas encore formellement démontré. La première étape consiste certainement à établir quels outils d’évaluation de la fonction rénale sont le mieux corrélés au débit de filtration glomérulaire pour définir précisément le stade de la néphropathie (cf tableau ci-dessous).

Classification de l’insuffisance rénale chronique selon la National Kidney Foundation

***Même si des recommandations concernant le dépistage et le suivi de la fonction rénale ont été élaborées récemment, aucune des méthodes de mesure du débit de filtration glomérulaire existante n’a été validée dans la population des patients infectés par le VIH qui diffère de la population générale par une dénutrition et des modifications de la composition corporelle fréquentes.***

La Société Américaine de Maladies Infectieuses a publié en 2005 les premières recommandations de prise en charge de la fonction rénale chez le sujet infecté par le VIH (Gupta, Eustace et al. 2005). Celles-ci ont été confirmées et augmentées au cours d’une conférence de consensus (à laquelle notre équipe a participé) réunissant les experts des KDIGO (Kidney Disease : Improving Global Outcome), groupe d’experts indépendants formé en 2003 dont la mission est « d’améliorer les pratiques de soins et l’évolution des patients atteints d’IRC en favorisant la collaboration, la coordination et la mise en place d’initiatives dédiées à l’application de recommandations cliniques pratiques » (Levey, Atkins et al. 2007). Ces recommandations soulignent l’importance de l’évaluation de la fonction rénale au diagnostic et de façon annuelle pour les patients à risque rénal s’il n’y a pas de protéinurie. Les patients présentant une protéinurie (plus d’une x à la bandelette) ou un débit de filtration glomérulaire estimé inférieur à 60 ml/min/1.73 m^2^ doivent être adressés à un néphrologue pour un bilan diagnostique.

L’évaluation de la fonction rénale doit être réalisée suivant ces recommandations, par le dosage de la créatinine si la masse musculaire est normale et grâce à des formules d’estimation du DFG dans les autres cas. Aucune formule ne peut être formellement conseillée car aucune n’est validée dans cette population, mais les auteurs proposent d’utiliser le Cockcroft et Gault dans un objectif d’adaptation des prescriptions à la fonction rénale car les études conduisant aux recommandations utilisent la plus part du temps cette formule (Rule, Cohen et al. 2007; Winston 2007).

Ces recommandations sont une aide à la pratique mais aucune étude ne documente la validité respective des différentes méthodes de mesure ou d’estimation du débit de filtration glomérulaire chez le patient infecté par le VIH. Or cette population présente de façon fréquente des anomalies de la composition corporelle avec une baisse significative de la masse musculaire chez les patients infectés par le VIH comparés à des patients séronégatifs. Cette différence semble plus marquée chez les caucasiens que chez les Afro Américains(Visnegarwala, Shlay et al. 2007). De plus, les syndromes de lipodystrophie/atrophie s’accompagnent d’une diminution de la masse grasse sous cutanée généralisée ou non, avec accumulation graisseuse dans des sites particuliers ou non et contribuent à la modification de la composition corporelle de ces patients. Enfin, la dénutrition n’est pas rare dans le cadre de l’infection par le VIH même si les syndromes de cachexie autrefois fréquents dans les stades évolués de la maladie sont aujourd’hui moins souvent rencontrés.

***Le patient infecté par le VIH fait donc partie d’une sous population dans laquelle aucun des marqueurs non invasifs du DFG n’est réellement pertinent, comme c’est le cas pour les femmes enceintes, les sujets cirrhotiques ou les grands obèses.***

Il paraît donc indispensable de définir quel marqueur simple permet au mieux d’évaluer le DFG chez le patient infecté par le VIH. Notre étude est la première comparant les marqueurs non invasifs à la mesure de référence se donnant ainsi le moyen de préciser vraiment leur pertinence. De nombreuses études se contentent de comparer l’intérêt du dosage de créatininémie à l’utilisation des formules d’estimation du DFG. Celles-ci reposant sur la créatininémie sont toutes empreintes d’une erreur potentielle liée aux modifications de la masse musculaire chez le sujet infecté par le VIH. Enfin, la cystatine C qui a l’avantage de ne pas être affectée par les modifications du poids ou de la masse musculaire est un marqueur qui semble intéressant pour évaluer la fonction rénale dans certaines populations. Très peu d’études ont évalué l’intérêt de la cystatine C dans cette population. Pourtant, la cystatine C s’est montré intéressante dans d’autres populations ou la masse musculaire est modifiée comme chez les cirrhotiques.

Enfin, les conséquences de l’évaluation correcte de la fonction rénale sont majeures chez ces patients en raison du nombre important de thérapeutiques auquel ils sont exposés et aux impératifs d’efficacité antirétrovirale qui reposent sur l’adaptation correcte des doses d’antirétroviraux à la fonction rénale (pour certains d’entre eux).

L’évaluation de la fonction rénale est habituellement réalisée sur la base du dosage de la créatininémie. Malheureusement, ce marqueur biologique qui présente l’avantage d’être peu coûteux et ne faisant pas l’objet d’un cycle nycthéméral présente néanmoins beaucoup d’inconvénients :

Son dosage en routine n’a pas fait l’objet d’une standardisation, la technique de référence IDMS pour « isotope dilution mass spectrometry » n’étant pas utilisée en routine car trop coûteuse.

La créatinine est censée être le reflet de la filtration glomérulaire, paramètre essentiel de la mesure de la fonction rénale lié au fonctionnement glomérulaire et la création de l’urine primitive. Malheureusement, le taux plasmatique de la créatinine est intimement lié à la masse musculaire puisqu’il s’agit d’une protéine dérivée du métabolisme de la créatine, protéine du muscle. Elle est donc également très dépendante de l’âge et du sexe, qui font varier considérablement la masse musculaire. Toutes les situations dans lesquelles la masse musculaire ne correspond pas à celle attendue pour l’âge et le sexe expose à de mauvaises interprétations du débit de filtration glomérulaire sur la base de la créatinine. Par ailleurs, le calcul de la clairance de la créatinine comme mesure du DFG est rendu imprécis par l’existence d’une réabsorption partielle de la créatinine et surtout d’une sécrétion tubulaire surtout importante lorsque la fonction rénale diminue. L’utilisation d’une technique de clairance pour la mesure du DFG repose sur le fait que le marqueur utilisé n’est ni réabsorbé ni sécrété par le tubule. La créatininémie n’est pas non plus un marqueur optimal pour la mesure de la clairance de la créatinine. Enfin, la clairance de la créatinine mesurée est rarement exacte étant sous la dépendance du recueil des urines de 24 heures exceptionnellement complet.

Des formules mathématiques d’estimation du DFG prenant en compte certains paramètres de variabilité de la fonction rénale ont été élaborées à partir de la créatinine dont celle de Cockcroft et Gault (CG) (1976) et celle de MDRD pour « Modification of Diet in Renal Disease ». La formule de CG inclut l’âge, le sexe, la masse pondérale dans son calcul. Elle a été déterminée dans une population masculine de 249 patients hospitalisés dont la fonction rénale était normale. Elle a été ensuite extrapolée à la population féminine. Elle surestime le DFG globalement d’environ 2 ml/min et doit être théoriquement ajustée à la surface corporelle. La formule de MDRD tient compte de l’âge et du sexe et introduit un facteur de correction pour les patients d’origine afro-américaine. Elle est ajustée à la surface corporelle. Elle sous estime globalement le DFG surtout chez les patients à fonction rénale normale et ne devrait en théorie pas être utilisée si le DFG attendu est supérieur à 60 ml/min/1.73m^2^. Elle a d’ailleurs été validée initialement dans une population de patients de DFG < 60 ml/min/1.73m^2^. Elle a ensuite été ré-évaluée en utilisant une technique de dosage de la créatinine calibrée par rapport à l’IDMS. La formule MDRD ré-exprimée après utilisation de techniques de dosage de la créatinine, utilisables en routine et calibrées par rapport à la technique de référence est désormais recommandée pour améliorer la pratique.

Dans l’ensemble, ces formules d’estimation du DFG n’ont pas été validées pour les patients infectés par le VIH (alors que de nombreuses études ont validé leur intérêt chez le sujet diabétique, cirrhotique, aux âges extrêmes etc..) alors que la masse musculaire de ces patients est variable et peut être modifiée par la maladie et les complications de la maladie et du traitement. Les caractéristiques du métabolisme de la créatine chez les sujets infectés par le VIH ne sont pas connues, alors que d’importantes anomalies du métabolisme musculaire sont présentes chez les patients lipodystrophiques comme l’accumulation musculaire des graisses en rapport avec une altération sévère de l’oxidation des lipides, ou une dysfonction musculaire liée à des anomalies mitochondriales.

D’autres méthodes d’estimation du DFG sont disponibles. La cystatine C, polypeptide basique de 13 kD (faible poids) est librement filtrée par le glomérule grâce à sa taille et sa charge positive. Elle est réabsorbée par les cellules tubulaires proximales et totalement catabolisée. Sa clairance est intimement corrélée au DFG mesuré par la clairance du Chrome EDTA (l’une des méthodes de référence). Elle appartient à la superfamille des cystatines et est synthétisée et sécrétée par toutes les cellules nucléées humaines. Elle n’est pas significativement influencée par l’âge et le sexe, l’inflammation. Son taux sérique augmente considérablement dans l’insuffisance rénale et elle a été proposée comme marqueur endogène du DFG. L’un de ses avantages est qu’une diminution modérée du DFG est associée avec une augmentation importante de son taux sérique. Son intérêt a été étudié par plusieurs études chez le sujet âgé, diabétique, transplanté. Aucune étude ne documente son intérêt chez le sujet infecté par le VIH. Il faut noter qu’une étude a mis en évidence le fait que les variations de la masse musculaire peuvent influencer le dosage de la cystatine C.

*La fréquence de l’altération de fonction rénale chez le sujet infecté par le VIH, justifiant l’élaboration de recommandations diagnostiques par l’Infectious Disease American Association en 2005, rend indispensable notre travail dont l’objectif est de clarifier la validité des différentes méthodes disponibles d’évaluation du DFG dans la population des patients infectés par le VIH. La mesure précise du DFG chez ces patients permet non seulement de proposer une prise en charge adaptée mais également d’améliorer la prescription des antirétroviraux. Il a en effet été démontré que les erreurs de prescription pour les antirétroviraux étaient fréquentes et responsables au moins dans deux études d’un impact significatif sur la survie des patients par l’intermédiaire d’un moins bon contrôle de la maladie.*

**Définition des objectifs du projet**

**Objectif principal** : Le but de cette étude est de déterminer quel est le ***paramètre le plus adapté permettant d’évaluer la fonction*** rénale des patients infectés par le VIH en comparant la pertinence de marqueurs biochimiques actuellement utilisés (créatinine) et des formules d’estimation du débit de filtration glomérulaire, à la cystatine C et à la mesure de référence réalisée en explorations fonctionnelles rénales par la méthode du Chrome EDTA.

**Objectif secondaire** : Le rôle de la ***technique de dosage de la créatinine*** et l’intérêt de l’harmonisation des méthodes de dosage sera étudié.

**Objectif secondaire** : L’impact des modifications de la ***composition corporelle*** (masse musculaire, masse maigre) sur la mesure du débit de filtration glomérulaire sera également évalué, permettant de préciser quel est l’outil idéal de mesure du DFG en fonction de la composition corporelle.

**Méthodologies proposées**

*a. L’organisation de la recherche.*

**Etude statistique :**

Le nombre de sujets inclus sera de 60.

On cherche à montrer que la clairance estimée ne diffère pas de la mesure de référence. Au vu de l’article de Mussap et al (Kidney Int 2002) sur des patients diabétique, on peut considérer qu’un différence de plus de 3 entre les deux clairance conduirait à ne pas pouvoir conclure à l’équivalence. D’après cet article on peut estimer à 20 l’écart type des différences. Dans notre étude nous avons prévu de restreindre les critères d’inclusion aux hommes caucasiens ayant une clairance entre 30 et 60 mL/min/1.73 m^2^ afin de diminuer la variabilité. Un écart type des différences nous permettra d’avoir une puissance de 81 % avec un effectif de 60 sujets (alpha = 5 %).

Le recrutement s’effectuera sur 12 mois ce qui paraît réaliste compte tenu du nombre de patients infectés par le VIH suivis dans le service et de la prévalence de l’insuffisance rénale chronique.

Pour chaque marqueur utilisé, pour chaque formule, pour chaque technique de dosage, les analyses suivantes seront réalisées :

- Calcul du coefficient de corrélation et de son intervalle de confiance entre la méthode de référence (DFG au Chrome EDTA) et la méthode testée ;
- Pour les formules calculant la clairance (MDRD, Cockcroft ainsi que les formules basées sur la cystatine C), calcul du coefficient de corrélation intra-classe et de son intervalle de confiance ;
- Pour les formules estimant la clairance, elles seront comparées en calculant la différence entre la valeur estimée et la valeur mesurée par la méthode de référence. Les différences seront alors comparées entre elles par un test de non infériorité de comparaison de moyenne apparié.
- Une analyse de l’influence de l’âge, du poids, et de la masse musculaire et de la masse maigre sur les mesures de la fonction rénale sera réalisée. Des modèles simples (régression linéaire) mais aussi plus complexes seront testés.

**Critères d’inclusion :**

Age supérieur à 18 ans

Infection par le VIH de type 1 confirmée (en Western Blot en plus de la sérologie positive) et DFG estimé par la formule de Cockcroft ou MDRD compris entre 30 et 60 ml/min/1.73 m^2^.

Consentement éclairé et écrit

Sujet de sexe masculin

Sujet caucasien

**Critères d’exclusion :**

Age inférieur à 18 ans

Absence de suivi possible (en rapport avec par exemple un passage en France).

Insuffisance rénale aiguë

Patient non affilié à un régime de sécurité sociale

Dysthyroïdie

Corticothérapie

**Anonymisation :**

L’anonymisation des patients se fera au moment de l’inclusion par le médecin responsable de l’inclusion par le biais d’un identifiant comportant :

Le numéro du service

Le numéro du patient, attribué dans l’ordre d’inclusion dans le service

Un code de 4 lettres, attribué de façon aléatoire et irréversible, en fonction des 2 premières lettres du nom et du prénom du patient (mode d’emploi = voir annexe)

Le schéma global de l’identifiant sera donc :

**|_0_|_0_|_1_| |_0_|_1_| |_X_|_X_|_X_|_X_|**

***N° service N° patient dans l’ordre d’inclusion Code lettres patient***

Cet identifiant sera généré par le médecin investigateur, qui conservera, dans son centre, les correspondances avec les données nominatives du patient. Le médecin responsable de l’inclusion remplira, après réalisation des examens de l’étude, le cahier d’observation qui sera anonyme et transmis sans l’identité du patient pour analyse des données à l’assistante de recherche clinique.

**Réalisation des prélèvements**

Les prélèvements sanguin et urinaire seront effectués au moment de la 2^ème^ consultation de Néphrologie. Un tube de sang de 5ml prélevé sur héparinate de lithium servira au dosage de la créatinine, de l’urée, de la CRP et de l’albumine. Un tube de 5ml prélevé sans anticoagulant servira au dosage de la cystatine C et à la constitution de la sérothèque. Un échantillon d’urines sera prélevé et constituera l’urinothèque. Les échantillons de sérum et d’urine seront congelés le jour du prélèvement dans le service de Biochimie du groupe hospitalier Pitié Salpétrière

La mesure du DFG nécessitera 3 prélèvements de 5 ml chacun après pose d’une voie d’abord veineuse (tube hépariné).

Enfin, la mesure de la composition corporelle (et plus particulièrement de la masse musculaire) sera réalisée par DEXA scan et ne nécessite pas de prélèvement.

Transcription des données dans le cahier d’observation

Toutes les informations requises par le protocole doivent être fournies dans le cahier d’observation et une explication donnée par l’investigateur pour chaque donnée manquante.

Les données devront être transférées dans les cahiers d'observation au fur et à mesure qu'elles sont obtenues qu'il s'agisse de données cliniques ou para-cliniques. Les données devront être copiées de façon nette et lisible à l'encre noire dans ces cahiers (ceci afin de faciliter la duplication et la saisie informatique).

Les données erronées dépistées sur les cahiers d'observation seront clairement barrées et les nouvelles données seront copiées sur le cahier avec les initiales et la date par le membre de l'équipe de l'investigateur qui aura fait la correction.

L'anonymat des sujets sera assuré par un numéro de code et les initiales de la personne qui se prête à la recherche sur tous les documents nécessaires à la recherche, ou par effacement par les moyens appropriés des données nominatives sur les copies des documents source, destinés à la documentation de la recherche.

Les données informatisées sur un fichier seront déclarées à la CNIL selon la procédure adaptée au cas.

Droit d'accès aux données et documents source

Les personnes ayant un accès direct conformément aux dispositions législatives et réglementaires en vigueur, notamment les articles L.1121-3 et R.5121-13 du code de la santé publique (par exemple, les investigateurs, les personnes chargées du contrôle de qualité, les moniteurs, les assistants de recherche clinique, les auditeurs et toutes personnes appelées à collaborer aux essais) prennent toutes les précautions nécessaires en vue d'assurer la confidentialité des informations relatives aux médicaments expérimentaux, aux essais, aux personnes qui s'y prêtent et notamment en ce qui concerne leur identité ainsi qu’aux résultats obtenus. Les données collectées par ces personnes au cours des contrôles de qualité ou des audits sont alors rendues anonymes.

1. *Les méthodes utilisées.*

**Dosage de créatinine :**

Le dosage de la créatinine plasmatique sera effectué par deux techniques. La technique colorimétrique dite de Jaffé compensée de la société Roche Diagnostics est la technique utilisée en routine par les laboratoires de Biochimie de l’hôpital Pitié-Salpétrière. La technique enzymatique, de la société Roche Diagnostics, a été standardisée par rapport à la technique de référence, la spectrométrie de masse avec dilution isotopique et permettra d’évaluer les formules de calcul selon les recommandations actuelles (Levey AS Ann Int Med 2006).

**Dosage de la cystatine C :**

La cystatine C sera mesurée sur les sérums des patients après décongélation de la sérothèque. La technique de dosage est une méthode immuno-néphélémétrique utilisant des anticorps de lapin, de la société Dade-Behring, la seule à avoir été approuvée par la FDA.

**Estimation du DFG :**

Le DFG sera estimé par les formules actuellement utilisées en routine c'est-à-dire la formule de Cockcroft et Gault, la formule de MDRD et la formule de MDRD simplifiée.

**Explorations fonctionnelles rénales :**

La mesure du DFG par la clairance de l’EDTA-^51^Cr (méthode choisie comme référence) sera effectuée dans le Service Central de Médecine Nucléaire de l’Hôpital Pitié Salpétrière. Elle nécessite l’injection d’un très faible quantité de ce traceur radioactif : l’activité injecté est de l’ordre de 3,5 MBq en IV. Un cathéter veineux est mis en place sur le bras opposé et trois prélèvements sanguins de 5 ml devront être effectués sur place respectivement 3 h, 4h et 5h après injection du traceur. Ces prélèvements seront ensuite centrifugés au laboratoire pour en extraire un échantillon plasmatique dont l’activité est mesurée dans un compteur puit. Le calcul de la clairance est effectué par 2 méthodes "simplifiées" (par rapport à la mesure compartimentale sur 12 prélèvements) : celle de Christensen et Groth (Christensen et Groth 1986) et celle de Bröcher-Mortensen (Bröcher-Mortensen 1972).

Sur le plan dosimétrique, la dose effective équivalente pour un adulte de 70 kg résultant d'une activité injectée de 3,5 Mbq se situe entre 0.008 mSv dans le cas d’une fonction rénale normale et 0.018 mSv pour une fonction rénale anormale (hypothèse d’augmentation d’un facteur 10 de la demi vie dans l’organisme et d’un facteur 4 du temps de transit rénal). L’irradiation occasionnée par ce dosage est donc très faible de l’ordre du centième de la dose effective moyenne annuelle liée à l’irradiation naturelle. Les principaux organes cibles sont la paroi vésicale (avec une dose absorbée de l’ordre de 2,3 10^-2^ mGy / Mbq), l’uterus (2,8 10^-3^ mGy / Mbq) et le rein (1,8 10^-3^ mGy / Mbq).

**Absorbtiométrie biphotonique :**

La mesure de la masse musculaire sera réalisée par une mesure du corps entier, avec composition corporelle, par la technique d’absorptiométrie biphotonique à rayons X (DXA).

Une seule mesure sera réalisée dans le service de rhumatologie de la Pitié par Dr Inaoui sur l’appareil LUNAR. La mesure est réalisée entre 20 et 30 minutes, en consultation externe.

Cette technique non invasive et très peu irradiante (de l’ordre de 0.4 µGy : irradiation inférieure à l’irradiation naturelle quotidienne) est une technique validée pour la mesure de la composition corporelle. Elle permet de quantifier la masse grasse, la masse maigre (= masse musculaire évaluée en gramme ou %) ainsi que le % de graisse dans différentes sous régions du corps ainsi que le corps entier.

1. **Echéancier de la recherche**

La période d’inclusion devra s’étendre sur 12 mois. Les dosages seront réalisés en une seule ou plusieurs fois mais de façon groupée.

*Modalités de recueil du consentement ou de la non-opposition*

Les patients seront recrutés au moment de leur consultation dans le service de Néphrologie de l’hôpital Pitié-Salpétrière. A ce moment là, les consentements éclairés des patients seront obtenus et le jour de leur consultation suivante avec le néphrologue, sera pris un rendez vous dans le service de rhumatologie de la Pitié Salpétrière où ils auront une mesure de la masse corporelle par absorptiométrie biphotonique. Les prélèvements sanguins et urinaires seront effectués au moment de cette deuxième consultation de Néphrologie. La mise en biothèque se fera le même jour dans le service de Biochimie de l’hôpital Pitié Salpétrière. Les dosages de cystatine C et de créatinine enzymatique ainsi que l’albuminémie et l’urée seront réalisés dans le service de Biochimie métabolique de l’hôpital Pitié-Salpétrière. La mesure de la filtration glomérulaire, effectuée dans le service de Médecine Nucléaire de l’hôpital Pitié Salpétrière, sera faite dans la semaine où auront lieu la consultation de Néphrologie et la mesure de la masse corporelle.

Conformément aux bonnes pratiques cliniques et aux dispositions légales en vigueur, tous les patients inclus seront préalablement informés par leur médecin référent des objectifs de l’étude, de sa méthodologie, de sa durée, de ses bénéfices, de ses contraintes et de l’avis du CPP. Ils devront prendre connaissance de la « lettre d’information » et signer le « consentement éclairé» donnant leur accord de participation à l’étude (cf annexes).

*Mise en biothèque*

Elle sera nécessaire car les dosages des marqueurs de la filtration glomérulaire à évaluer seront effectués en une seule fois sur les sérums congelés pour éviter la variabilité technique inhérente à l’utilisation d’anticorps pour le dosage de cystatine C.

1. **Perspectives du projet**

La comparaison de la validité des différents marqueurs versus la mesure de référence permettra de formuler des conseils précisant quel est le marqueur non invasif le mieux corrélé au DFG suivant l’âge, le poids, la masse musculaire et le niveau de fonction rénale des patients.

1. **Engagement à présenter le projet au CPP**

Le promoteur est défini par la loi 2004-806 du 9 août 2004. Dans cette recherche, l'AP-HP sera le promoteur et le Département de la Recherche Clinique et du Développement (DRCD) en assurera les missions réglementaires.

*Demande d’avis au comite de protection des personnes*

En accord avec l'article L.1123-6 du Code de Santé Publique, le protocole de recherche sera soumis par le promoteur à un Comité de Protection des Personnes L'avis de ce comité sera notifié à l’autorité compétente par le promoteur avant le démarrage de la recherche.

*Modifications*

Le DRCD devra être informé de tout projet de modification du protocole par l’investigateur coordonnateur.

Les modifications devront être qualifiées en substantielles ou non.

Une modification substantielle est une modification susceptible, d'une manière ou d'une autre, de modifier les garanties apportées aux personnes qui se prêtent à la recherche biomédicale (modification d’un critère d’inclusion, prolongation d’une durée d’inclusion, participation de nouveaux centres,…).

Après le commencement de la recherche, toute modification substantielle de celle-ci à l’initiative du promoteur doit obtenir, préalablement à sa mise en oeuvre, un avis favorable du comité et une autorisation de l’autorité compétente. Dans ce cas, si cela est nécessaire, le comité s’assure qu’un nouveau consentement des personnes participant à la recherche est bien recueilli.

Par ailleurs, toute extension de la recherche (modification profonde du schéma thérapeutique ou des populations incluses, prolongation des traitements et ou des actes thérapeutiques non prévus initialement dans le protocole) devra être considérée comme une nouvelle recherche.

**Bibliographie**

*Brochner-Mortensen J (1972)” A simple method for determination of glomerular filtration rate” Scand. J. Clin. Lab. Invest.* ***30*** *: 271-274.*

*Cheung, C. Y., K. M. Wong, et al. (2007). "Prevalence of chronic kidney disease in Chinese HIV-infected patients." Nephrol Dial Transplant.*

*Choi, A., R. Rodriguez, et al. (2006). HIV antiretroviral therapy in chronic kidney disease. American Society of Nephrology, San Diego, USA.*

*Christensen A.B., Groth S. (1986) “Determination of Tc99m-DTPA clearance by a single plasma sample method Clin. Physiol 6 : 579-588.*

*Crane, H. M., S. E. Van Rompaey, et al. (2006). "Antiretroviral medications associated with elevated blood pressure among patients receiving highly active antiretroviral therapy." Aids* ***20****(7): 1019-26.*

*Franceschini, N., S. Napravnik, et al. (2005). "Incidence and etiology of acute renal failure among ambulatory HIV-infected patients." Kidney Int* ***67****(4): 1526-31.*

*Gebo, K. A. (2006). "HIV and aging: implications for patient management." Drugs Aging* ***23****(11): 897-913.*

*Gupta, S. K., B. W. Mamlin, et al. (2004). "Prevalence of proteinuria and the development of chronic kidney disease in HIV-infected patients." Clin Nephrol* ***61****(1): 1-6.*

*Gupta, S. K., J. A. Eustace, et al. (2005). "Guidelines for the management of chronic kidney disease in HIV-infected patients: recommendations of the HIV Medicine Association of the Infectious Diseases Society of America." Clin Infect Dis* ***40****(11): 1559-85.*

*Hiramatsu, N., K. Hiromura, et al. (2007). "Angiotensin II type 1 receptor blockade inhibits the development and progression of HIV-associated nephropathy in a mouse model." J Am Soc Nephrol* ***18****(2): 515-27.*

*Jung, O., M. Bickel, et al. (2004). "Hypertension in HIV-1-infected patients and its impact on renal and cardiovascular integrity." Nephrol Dial Transplant* ***19****(9): 2250-8.*

*Kimmel, P. L., G. J. Mishkin, et al. (1996). "Captopril and renal survival in patients with human immunodeficiency virus nephropathy." Am J Kidney Dis* ***28****(2): 202-8.*

*Krawczyk, C. S., S. D. Holmberg, et al. (2004). "Factors associated with chronic renal failure in HIV-infected ambulatory patients." Aids* ***18****(16): 2171-8.*

*Levey A.S., Coresh J., Greene T., Stevens L.A., Yaping L.Z., Hendriksen S., Kusek J.W., Van Lente F. (2006). “Using standardized serum creatinine values in the modification of diet in renal disease equation for estimating glomerular filtration rate”Ann. Intern.Med****.145****:247-254.*

*Levey, A. S., R. Atkins, et al. (2007). "Chronic kidney disease as a global public health problem: Approaches and initiatives - a position statement from Kidney Disease Improving Global Outcomes." Kidney Int* ***72****(3): 247-59.*

*Locatelli, F., D. Marcelli, et al. (1996). "Proteinuria and blood pressure as causal components of progression to end-stage renal failure. Northern Italian Cooperative Study Group." Nephrol Dial Transplant* ***11****(3): 461-7.*

*Mocroft, A., O. Kirk, et al. (2007). "Chronic renal failure among HIV-1-infected patients." Aids* ***21****(9): 1119-27.*

*Morlat, P., E. Déti, et al. (2007). Prevalence of renal failure in HIV-infected patients and associated factors. IAS Conference of HIV pathogenesis, treatment and prevention. Sydney, Australia.*

*Parving, H. H., H. Lehnert, et al. (2001). "The effect of irbesartan on the development of diabetic nephropathy in patients with type 2 diabetes." N Engl J Med* ***345****(12): 870-8.*

*Rule, A. D., S. D. Cohen, et al. (2007). "Editorial comment: screening for chronic kidney disease requires creatinine references ranges not equations." AIDS Read* ***17****(5): 262-3.*

*Seaberg, E. C., A. Munoz, et al. (2005). "Association between highly active antiretroviral therapy and hypertension in a large cohort of men followed from 1984 to 2003." Aids* ***19****(9): 953-60.*

*Shah, S. S., J. P. McGowan, et al. (2002). "Comorbid conditions, treatment, and health maintenance in older persons with human immunodeficiency virus infection in New York City." Clin Infect Dis* ***35****(10): 1238-43.*

*Szczech, L. A., S. K. Gupta, et al. (2004). "The clinical epidemiology and course of the spectrum of renal diseases associated with HIV infection." Kidney Int* ***66****(3): 1145-52.*

*Szczech, L., S. Gange, et al. (2002). "Predictors of proteinuria and renal failure among women with HIV infection." Kidney Int.* ***61****(1): 195-202.*

*Tourret, J., I. Tostivint, et al. (2006). "Outcome et prognosis factors in HIV-infected hemodialysis patients." Clinical Journal of the American Society of Nephrology(1): 1241-47.*

*Visnegarwala, F., J. C. Shlay, et al. (2007). "Effects of HIV infection on body composition changes among men of different racial/ethnic origins." HIV Clin Trials* ***8****(3): 145-54.*

*Wei, A., G. C. Burns, et al. (2003). "Long-term renal survival in HIV-associated nephropathy with angiotensin-converting enzyme inhibition." Kidney Int* ***64****(4): 1462-71.*

*Winston, J. A. (2007). "Assessing kidney function in HIV infection." AIDS Read* ***17****(5): 257-61, 264.*

*Wyatt, C. M., R. R. Arons, et al. (2006). "Acute renal failure in hospitalized patients with HIV: risk factors and impact on in-hospital mortality." Aids* ***20****(4): 561-5.*

*Yeni, P. (2006). "Prise en charge medicale des personnes infectées par le VIH, recommandations du groupe d'expert."*

**Marqueurs non invasifs de la fonction rénale chez le patient et infecte par le VIH**

**NOTE D’INFORMATION AU PATIENT ET CONSENTEMENT**

Madame, Monsieur,

Vous êtes suivi(e) médicalement en raison d’une infection par le *virus de* *l’immunodéficience humaine (VIH) et votre fonction rénale doit être précisément évaluée*. En effet, votre infection peut s’accompagner d’un retentissement sur la fonction de vos reins. Actuellement, la meilleure manière (la plus simple, la moins invasive, la moins coûteuse) de mesurer votre fonction rénale n’est pas formellement établie.

Nous vous sollicitons pour participer à une étude dont l’objectif est de déterminer quelle est la meilleure façon de mesurer la fonction rénale lorsque l’on est atteint d’une infection par le VIH.

Cette étude va permettre de comparer l’intérêt du dosage de la créatinine (réalisé sur une simple prise de sang), des formules mathématiques d’estimation du débit de filtration glomérulaire (à partir du dosage de créatinine) et du dosage de la cystatine C (réalisé sur la même prise de sang) par rapport à la méthode de référence de mesure de la fonction rénale suivant votre masse musculaire (une journée en hôpital de jour). Cette mesure de référence nécessite l'injection d'une très faible quantité d'un traceur radioactif mais dont l'irradiation procurée peut être considérée comme négligeable (moins d'un centième de la

dose moyenne annuelle occasionnée par l'irradiation naturelle). Elle permettra d’établir quelle est la méthode la plus fiable et la plus simple pour suivre votre fonction rénale.

Votre participation à cette étude se traduira par une mesure de votre masse corporelle dans le service de Rhumatologie et une journée d’hospitalisation dans le service de Médecine Nucléaire de l’Hôpital Pitié-Salpétrière au cours de laquelle sera réalisée une prise de sang après mise en place d’une perfusion veineuse périphérique. Le volume sanguin total prélevé est résumé ci-dessous :

| **Nombre de Prélèvements** | **Moment du prélèvement** | **Quantité prélevée (mL)** |
| --- | --- | --- |
| **2** | 2^ème^ Consultation de Néphrologie | 10 |
| **3** | Mesure de la filtration glomérulaire | 15 |

A l’issue de des prélèvements, les résultats des dosages seront communiqués, de façon anonyme, à notre centre de coordination, puis analysés.

Le fichier informatique utilisé pour réaliser la présente recherche a fait l’objet d’une demande d’autorisation à la CNIL (Commission Nationale Informatique et Libertés), en application des articles 40-1 et suivants de la loi « informatique et libertés ». Les données médicales vous concernant ne seront transmises qu’au promoteur et, le cas échéant aux autorités sanitaires habilitées, dans des conditions garantissant *leur confidentialité*. Vous pouvez exercer vos droits d’accès et de rectification auprès de votre médecin référent.

Le Comité d’Éthique (CPP) du Groupe Hospitalier Pitié-Salpêtrière (Paris) a donné un avis favorable le XX/XX/06 au déroulement de cette étude.

Le refus de participer à cette étude n’affectera en aucun cas le traitement de votre maladie. Vous êtes également en droit d’interrompre votre participation à tout instant, sans avoir à justifier votre décision. Cela ne remet pas en cause la qualité des soins dispensés ultérieurement.

Vous pourrez, si vous le souhaitez, être tenu informé des résultats globaux de la recherche une fois que celle-ci sera achevée.

Si vous avez d’autres questions concernant le déroulement de cet essai, veuillez contacter le

Dr ________________________  (Tel) : _________________________.

**A faire en 3 exemplaires**

**(1 pour le patient, 1 pour l’investigateur et 1 pour le promoteur)**

Je soussigné(e) ________________________________________________ certifie avoir lu et pris connaissance des modalités de déroulement de cette étude. J’ai été averti des bénéfices et des contraintes de cet essai. J’ai bien lu et compris les informations décrivant cette étude et j’ai eu la possibilité de poser toutes les questions qui me paraissaient utiles pour la bonne compréhension de la note d’information.

***J’accepte librement et volontairement de participer à cette étude, dans les conditions décrites ci-dessus.***

Mon consentement ne décharge pas les organisateurs de la recherche de leurs responsabilités et je conserve tous mes droits garantis par la loi.

Le Dr ___________________________ m’a clairement précisé que je suis libre d’accepter, de refuser ou d’interrompre à tout moment (en informant le médecin) ma participation à cette recherche. Cela ne remettra, en aucun cas, en cause la qualité des soins qui me seront dispensés ultérieurement.

J’accepte que les données recueillies à l’occasion de ce protocole puissent faire l’objet d’un traitement informatisé. J’ai pris connaissance de mon droit d’accès et de rectification des informations nominatives me concernant, conformément à la loi « Informatiques et Libertés » du 6 janvier 1978.

Toutes les données et informations qui me concernent resteront *STRICTEMENT CONFIDENTIELLES*. Je n’autorise leur consultation que par des personnes directement concernées par cette étude, ainsi que par le représentant des Autorités de Santé.

Le___/___/_____à _________________*Signature du patient*

Je soussigné Docteur ______________________ certifie avoir communiqué toute information utile concernant cette étude.

Le___/___/_____à _________________

*Signature du médecin*

**A faire en 3 exemplaires**

**(1 pour le patient, 1 pour l’investigateur et 1 pour le promoteur)**
