# Supplementary material for: Impact of lean mass and bone density on glomerular filtration rate estimation in people living with HIV/AIDS
Source: PLoS One. 2017 Nov 2;12(11):e0186410. doi: 10.1371/journal.pone.0186410 (PMC5668131; doi:10.1371/journal.pone.0186410)
Supplement: S2 File — (DOCX) [file pone.0186410.s002.docx]

# Markers of glomerular filtration rate in the HIV infected patient – Role of body composition

***Renal function assessment in HIV patient : HIVERS Study***

Sponsor or principal investigator : Pr Corinne Isnard Bagnis

Organization: Nephrology Department, Pitie Salpetriere Hospital, Paris, France.

Phone: +33(0) 1 42 17 72 27

Email: corinne.bagnis@psl.aphp.fr

Review board N° 79-08

CPP- Ile de France VI Groupe Pitie Salpetriere

Head of review board :

Name: Jean Louis Golmard

Business Phone: +33 42 16 16 83

Business Email: Jean-louis.golmard@psl.aphp.fr

Business Address: CPP IDF VI 47 Boulevard de l’Hôpital 75013 Paris

## Abstract

Recent progress in antiretroviral therapy has turned HIV infection into a chronic disease. Patients survival has dramatically improved but complications may occur that need to be prevented and monitored. As much as 10 % of HIV patients may suffer from chronic kidney disease, an affection that is not symptomatic until a very late stage secondary to HIV infection, drugs exposure, hypertension or diabetes. Guidelines have suggested that renal function should be regularly assessed in HIV patients to perform early diagnosis for chronic kidney disease and allow initiation of preventive measures aimed at preserving renal function.

Plasma creatinine dosage is the easiest way to evaluate renal function but glomerular filtration rate estimation from cockcroft or MDRD formulae is a much better indicator of renal function. Other markers like cystatin C may be used. None of these markers has been validated in HIV patients. Therefore our study is aimed at comparing validity of creatinine clearance estimation with Cockcroft and Gault and MDRD formula and cystatin C compared to the gold standard measurement of glomerular renal function.

*Aim of the study :* The aim of our study is to test the different available markers for the estimation of glomerular filtration rate (GFR) in HIV patients in order to determine which one is the more appropriated in this particular population. We will analyze the role of the variation in muscular mass in the estimation of GFR

*Background*: Several lines of evidence point to renal disease becoming an important complication of human immunodeficiency virus infection and therapy. The spectrum of renal disease in the HIV patient has dramatically changed with HIV associated nephropathy becoming less prevalent and chronic kidney disease [CKD] becoming an everyday concern parallel to the prevalence of renal risk factors such as aging, hypertension and diabetes. Different studies have shown that the prevalence of CKD defined as an estimated glomerular filtration rate below 60 ml/min/1.73m2 is around 10% in HIV patients. CKD is associated with increased mortality, a new concept that has emerged in the past years putting CKD in the top 5 critical cardiovascular risk factors. Evaluating individual renal function in HIV patients allows better care in prevention of cardiovascular events as well as initiation of nephroprotection strategies in order to slow down the loss of renal function and alleviate or postpone the need for dialysis. Since HIV patients also receive chronic multitherapy, evaluating renal function is also mandatory to allow drug dosage adaptation.

Plasma creatinine dosage is routinely used to estimate GFR but it is closely correlated to age, sex, weight and ethnic origin of the patients. All these factors make creatinine unreliable for GFR estimation. Laboratory dosage of creatinine may be performed with different techniques (Jaffé or enzymatic dosage) with a 20% variation in the results for the same patient. None of the GFR estimation formulae (Cockcroft et Gault or MDRD) have been validated in HIV patients. Cystatin C, a new marker still under evaluation, seems to show some interest in such patients because of its relative independency with regard to muscle mass, but has not been tested in HIV patients. Some cystatin C based formulae have been proposed but not yet tested and validated in HIV patients. HIV infected patients may exhibit abnormal body composition particularly affecting lipids but also, as shown recently, muscle mass. Our study will precisely measure body composition in order to allow interpretation of the variations of the renal markers with regard to muscle mass.

*Méthods*

60 patients (men, causasians) with an estimated GFR between 60 and 15 ml/min/1.73m^2^ will be included in the study. After giving all necessary information and collection of informed consent, blood will be drown to allow creatinine, cystatin C, albumin, C reactive protein and urea dosages. GFR will be measured using plasmatic clearance of EDTA-51Cr. Body composition will be performed using DEXA scan. Two visits will be necessary to complete the evaluation for every patient, scheduled at the same time than the routine medical visits.

Primary outcome : GFR estimated with Cockcroft and Gault and MDRD formulae and cystatin C dosage compared to isotopic evaluation of GFR (assessed within 10 weeks after inclusion)

Secondary outcome : Variability of creatinine plasma dosage within two different methods

Role of bone density on validity of renal function markers in HIV patients

Inclusion criteria :

- 18 Years and older

- Patients must have detectable HIV-1 by western-blot

consent signature

- Estimated glomerular filtration rate, by Modification of Diet in Renal Disease (MDRD) or Cockcroft equation, betwen 30 and 60 ml/min/1.73m2

- Male

- Caucasian

- Patient provides informed consent

- Patient able to respect the protocol

- Affilié à un régime de sécurité sociale

Exclusion criteria :

Patient with:

- acute renal failure

- dysthyroidy

metallic prosthesis

- unable to understand the informed consent document

- venous puncture impossible

- receiving steroids

- no possible follow up

*Expected results :*

Our study should allow better definition of the ideal marker for GFR in HIV infected male. Il will also afford better comprehension of the factors involved in the variation of creatinine dosage in this population namely those related to the biochemical dosage and to the change in body composition.

*Conclusion* : HIV infection and its treatment is definitely a serious risk factor for renal disease. Although, optimal care for HIV-infected patients becomes more and more complex due to multiple comorbidities, efforts must be made to diagnose a decline in glomerular filtration rate in order to provide improved control of CKD-associated cardiovascular risk and optimized antiretroviral therapy. Therefore evaluating glomerular filtration rate is critical in HIV-infected patients to allow determination of the optimum follow up strategy and the critical therapeutic measures, namely drug dosage adaptation.

Key words : HIV, glomerular filtration rate, cockcroft, MDRD formula, creatinine, cystatin C, isotopic clearance, EDTA-51Cr, DEXA scan;
