# Supplementary figures and images for: Impact of lean mass and bone density on glomerular filtration rate estimation in people living with HIV/AIDS
Source: PLoS One. 2017 Nov 2;12(11):e0186410. doi: 10.1371/journal.pone.0186410 (PMC5668131; doi:10.1371/journal.pone.0186410)

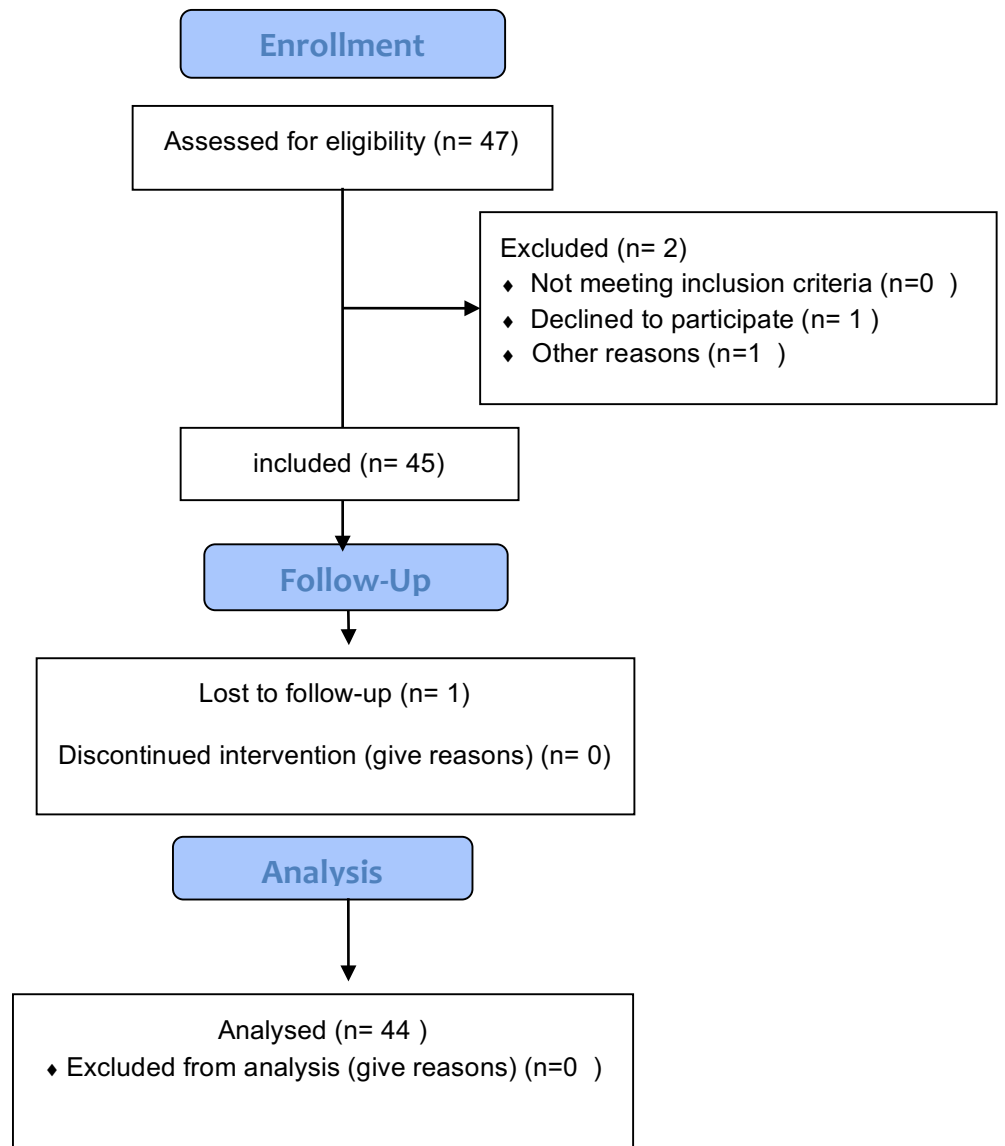

Supplement: S1 Fig — (PDF) [file pone.0186410.s004.pdf]

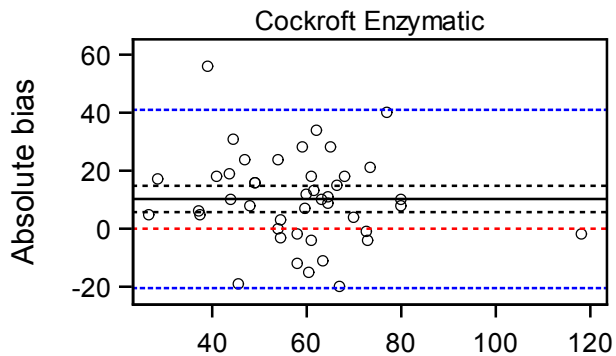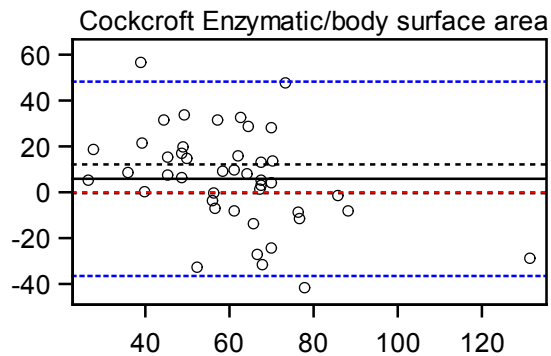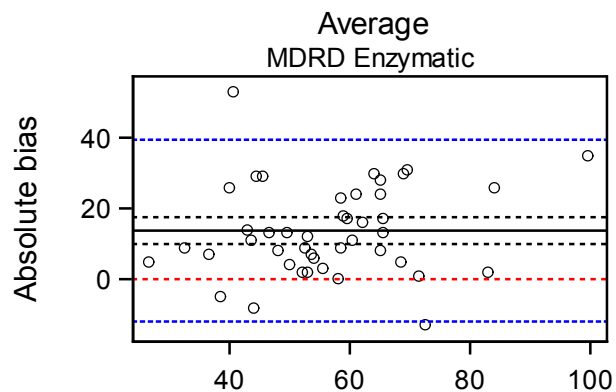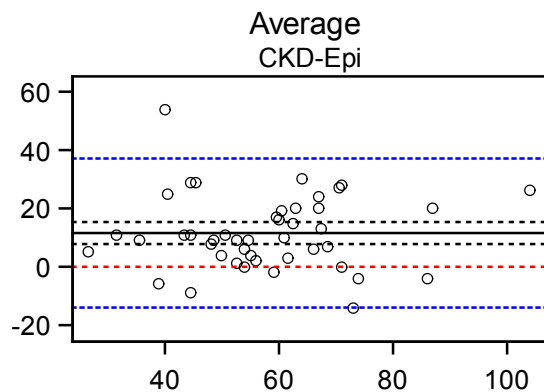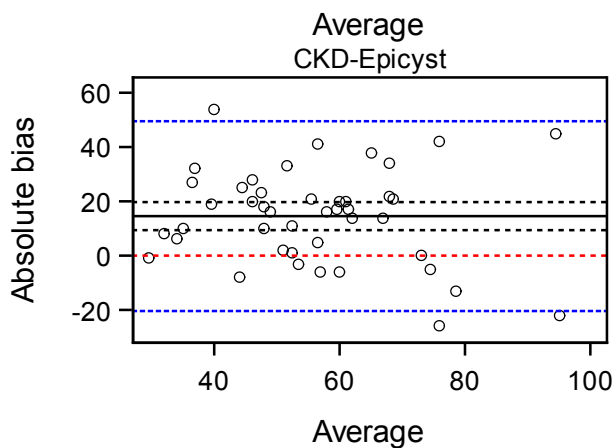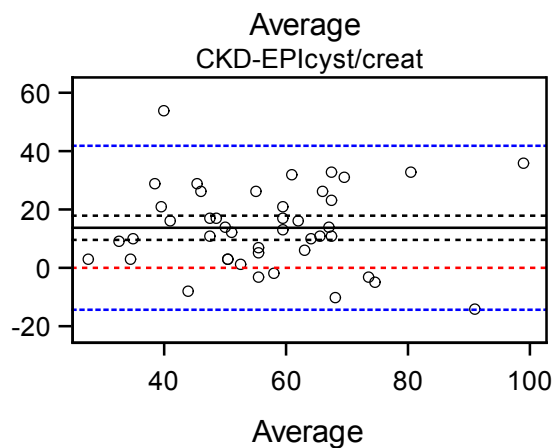

Supplement: S2 Fig — Accuracy: percentage of estimates more than 30% of measured GFR. (PDF) [file pone.0186410.s005.pdf]
