# Supplementary material for: Impact of lean mass and bone density on glomerular filtration rate estimation in people living with HIV/AIDS
Source: PLoS One. 2017 Nov 2;12(11):e0186410. doi: 10.1371/journal.pone.0186410 (PMC5668131; doi:10.1371/journal.pone.0186410)
Supplement: Supplementary file 5 [file pone.0186410.s006.docx]

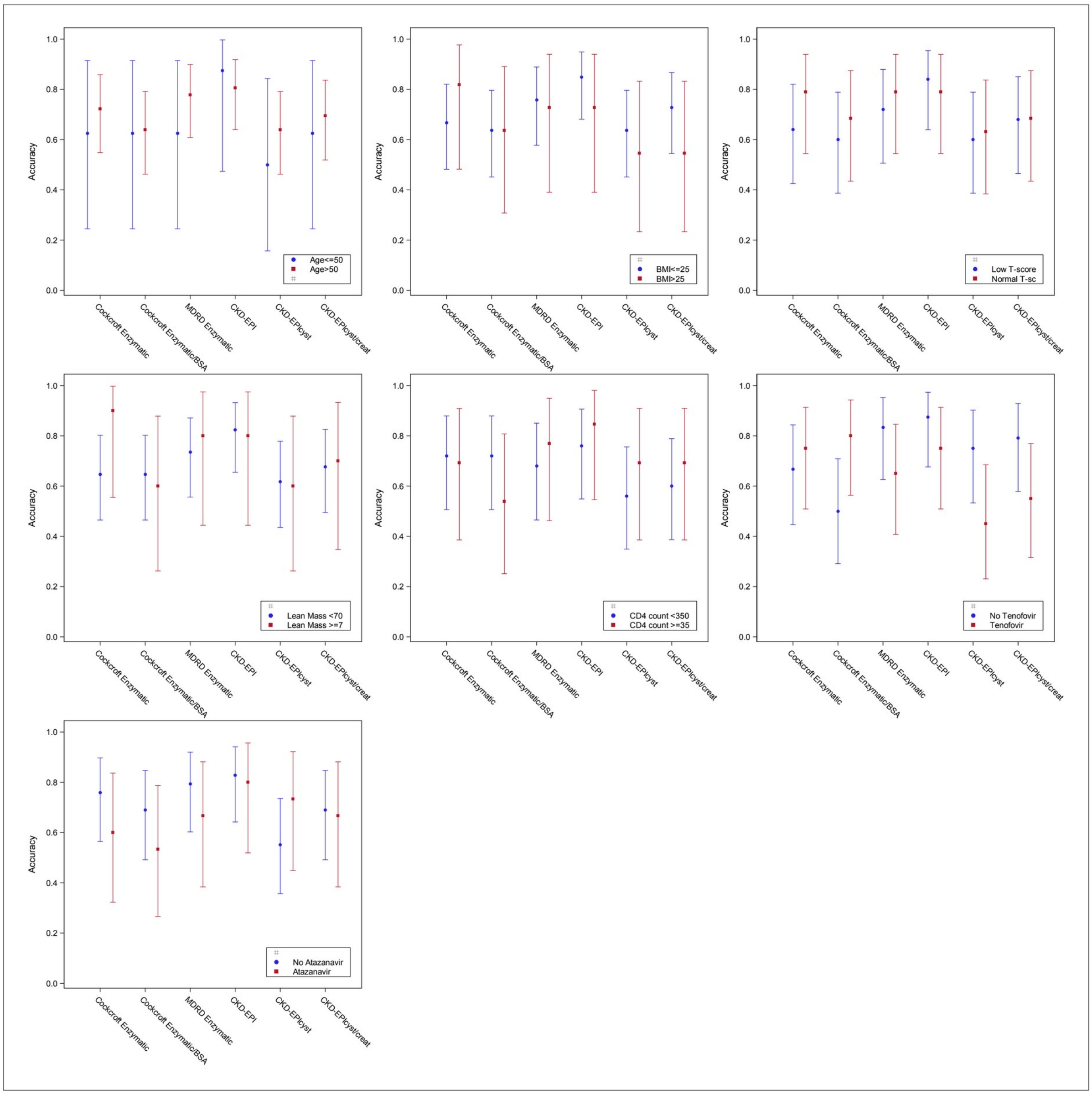

Accuracy
1.0
0.8
0.6
0.4
0.2
0.0
Cockcroft Enzymatic
Cockcroft Enzymatic/BSA
MDRD Enzymatic
CKD-EPI
CKD-EPIcyst
CKD-EPIcyst/creat
Age<=50
Age>50
BMI<=25
BMI>25
Low T-score
Normal T-sc
Lean Mass <70
Lean Mass >=7
CD4 count <350
CD4 count >=35
No Tenofovir
Tenofovir
No Atazanavir
Atazanavir
